# SXGBsite: Prediction of Protein–Ligand Binding Sites Using Sequence Information and Extreme Gradient Boosting

**DOI:** 10.3390/genes10120965

**Published:** 2019-11-22

**Authors:** Ziqi Zhao, Yonghong Xu, Yong Zhao

**Affiliations:** School of Electrical Engineering, Yanshan University, Qinhuangdao 066004, China

**Keywords:** protein–ligand binding site, SMOTE, Extreme Gradient Boosting, discrete cosine transform (DCT), discrete wavelet transform (DWT)

## Abstract

The prediction of protein–ligand binding sites is important in drug discovery and drug design. Protein–ligand binding site prediction computational methods are inexpensive and fast compared with experimental methods. This paper proposes a new computational method, SXGBsite, which includes the synthetic minority over-sampling technique (SMOTE) and the Extreme Gradient Boosting (XGBoost). SXGBsite uses the position-specific scoring matrix discrete cosine transform (PSSM-DCT) and predicted solvent accessibility (PSA) to extract features containing sequence information. A new balanced dataset was generated by SMOTE to improve classifier performance, and a prediction model was constructed using XGBoost. The parallel computing and regularization techniques enabled high-quality and fast predictions and mitigated overfitting caused by SMOTE. An evaluation using 12 different types of ligand binding site independent test sets showed that SXGBsite performs similarly to the existing methods on eight of the independent test sets with a faster computation time. SXGBsite may be applied as a complement to biological experiments.

## 1. Introduction

Accurate prediction of protein–ligand binding sites is important for understanding protein function and drug design [1,2,3,4]. The experiment-based three-dimensional (3D) structure recognition of protein–ligand complexes and binding sites is relatively expensive and time consuming [5,6]. Computational methods can predict binding sites rapidly and can be applied as a supplement to experimental methods. Structure-based methods, sequence-based methods, and hybrid methods are the commonly applied computation methods [7,8]. 

The structure-based methods are usually applied to predict ligand binding sites with known 3D protein structures [2,9,10,11]. We focused on the sequence-based method without 3D structure information, and only a few structure-based methods are listed due to the rapid update of these different methods. Pockets on the protein surface can be identified by computing geometric measures, such as LIGSITE^CSC^ [2,12], CASTp [13,14,15,16], LigDig [17], and Fpocket [18,19]. LIGSITE^CSC^ [2,12] identifies pockets through the number of surface–solvent–surface events and clusters. CASTp [13,14,15,16] locates and measures pockets on 3D protein structures and annotates functional information for specific residues. Unlike traditional protein-centric approaches, LigDig [17] is a ligand-centric approach that identifies pockets using information from PDB [20], UniProt [21], PubChem [22], ChEBI [23], and KEGG [24]. Fpocket [18,19] identifies pockets using structure-based virtual screening (SBVS). RF-Score-VS [25] improves the performance of SBVS and can be used in the open source ODDT [26,27]. FunFOLD [1] introduces cluster identification and residue selection to automatically predict ligand binding residues. CHED [28] constructs a model to predict metal-binding sites using geometric information and machine learning methods. The integration of sequence information in structure-based methods helps improve prediction performance [29,30,31]. ConCavity [29] integrates sequence evolution information and structure information to recognize pockets. COACH [30] and HemeBIND [31] construct prediction models and identify ligand binding sites using sequence and structural information features based on machine learning methods. In general, structure-based methods and hybrid methods enable high-quality predictions when 3D structures of protein–ligand complexes are known [8]. 

Sequence-based methods can predict protein–ligand binding sites with unknown 3D structures [5,32,33,34]. MetaDBSite [32] integrates six methods, including DISIS [35], DNABindR [36], BindN [37], BindN-rf [38], DP-Bind [39], and DBS-PRED [40], and produces better results than each of the methods alone. DNABR [5] introduces sequence characteristics based on the random forest method [41] to study the sequence characteristics that delineate the physicochemical properties of amino acids. Both SVMPred [33] and NsitePred [34] construct support vector machine (SVM) [42] prediction models using multiple features including position-specific scoring matrix (PSSM), predicted solvent accessibility (PSA), predicted secondary structure (PSS), and predicted dihedral angles. TargetS [7] considers the ligand-specific binding propensity feature and builds models using a scheme of under-sampling and ensemble SVMs. EC-RUS [8] selects position-specific scoring matrix discrete cosine transform (PSSM-DCT) and PSA as features, constructs prediction models using under-sampling and ensemble classifiers, and compares the prediction quality of weighted sparse representation based classifier (WSRC) [43] and SVM.

**Figure 1:**
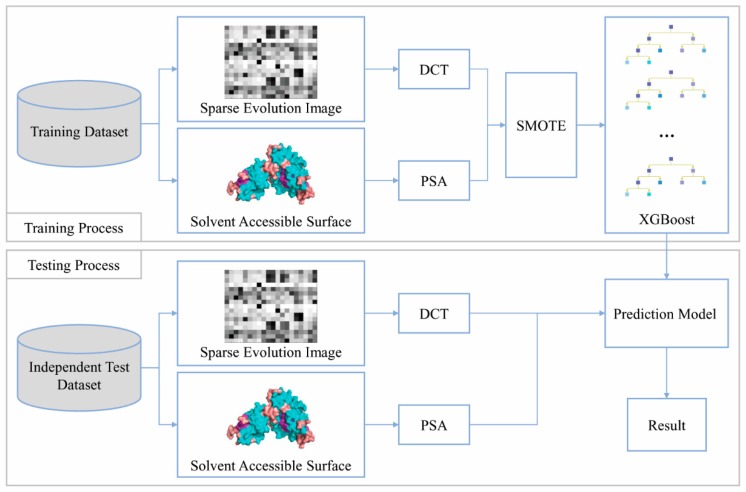
SXGBsite Flowchart. During the training process, the position-specific scoring matrix (PSSM) feature of residues was represented by the sparse evolution image, discrete cosine transform (DCT) compressed the PSSM feature to obtain the PSSM-DCT feature, and the predicted solvent accessibility (PSA) feature was used to improve the prediction quality. SMOTE generated a new balanced training set with the training set of PSSM-DCT + PSA features, and the prediction model of binding residues was constructed by the balanced training set and XGBoost. During the testing process, the unbalanced independent test set, which also extracted the PSSM-DCT + PSA features, was input into the prediction model to obtain the result.

One machine learning model in the ensemble classifiers is usually built with a dataset generated by under-sampling, and a new model is built after the end of the building process of the previous model. In this paper, this process is called the serial method, and performs well among the sequence-based methods at present but requires more computation time [8,44]. Here, we propose a new parallel method for predicting protein–ligand binding site residues using the evolutionary conservation information of homologous proteins. The main information source used for predictions is the PSSM of sequences. The prediction model of binding residues is constructed by XGBoost machine learning method [45] with the synthetic minority over-sampling technique (SMOTE) [46], and this method reduces the computation time while ensuring prediction quality. We compared the prediction qualities of different feature combination schemes of PSSM-DCT [8,47,48,49], PSSM-discrete wavelet transform (DWT) [49,50,51] and PSA [52], and PSSM-DCT + PSA scheme was selected. For the dataset imbalance problem, XGBoost with SMOTE was applied to construct the protein–ligand binding site prediction models, and the optimal parameters were determined by five-fold cross-validation and a grid search method. The models were validated on 12 different types of protein–ligand binding site datasets. The SXGBsite process is shown in Figure 1.

## 2. Materials and Methods 

### 2.1. Benchmark Datasets

The benchmark datasets were constructed based on the BioLip database [53] developed by Yu et al. [7], including the training and independent test datasets of 12 different ligands. The 12 types of ligands used were five types of nucleotides, five types of metal ions, DNA and Heme (Table 1). The source code and datasets are available at https://github.com/Lightness7/SXGBsite.

### 2.2. Feature Extraction

#### 2.2.1. Position-Specific Scoring Matrix

The position-specific scoring matrix (PSSM) encodes the evolution information of the protein sequence. The PSSM of each sequence was obtained using PSI-BLAST [54] in the database of non-redundant protein sequences (nr) with three iterations and the E-value of 0.001. The PSSM is a matrix of *L* × 20, where *L* rows represent *L* amino acid residues in the protein sequence, 20 columns represent the probability that each residue is mutated to 20 native residues, as follows:

(1) $$\mathrm{PSSM}=\left[\begin{array}{cccc} P_{1,1} & P_{1,2} & \cdots & P_{1,20}\\ P_{2,1} & P_{2,2} & \cdots & P_{2,20}\\ \vdots & \vdots & \vdots & \vdots \\ P_{L,1} & P_{L,2} & \cdots & P_{L,20} \end{array}\right]$$

**Figure 2:**
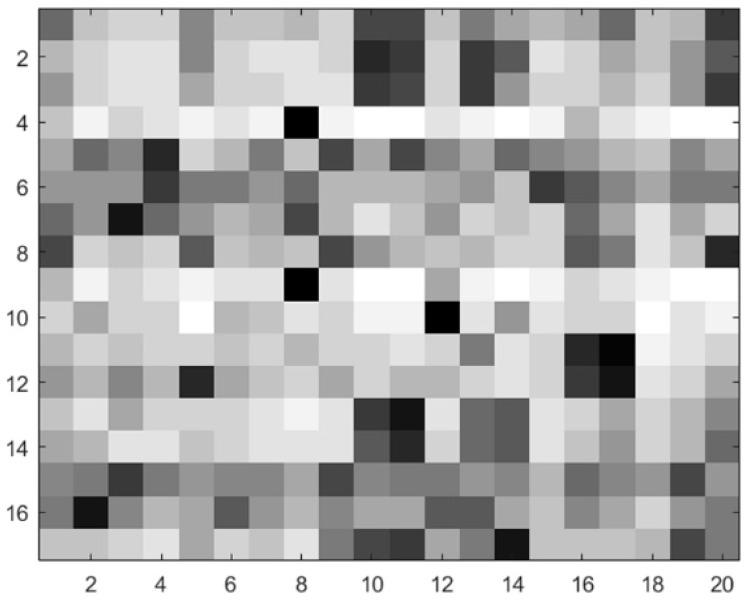
Residue sparse evolution image.

The PSSM feature of contiguous residues was extracted with a sliding window with size *w*. The window was centered on the target residue and contained (*w* − 1)/2 adjacent residues on both sides. The size of the PSSM feature matrix was *w* × 20, and the residue sparse evolution image [8,48] is shown in Figure 2. The window size *w* = 17 was selected after testing different values of *w*, and the dimensions of the PSSM feature were 17 × 20 = 340.

#### 2.2.2. Discrete Cosine Transform

Discrete Cosine Transform (DCT) [47] is widely applied for lossy data compression of signals and images. In this study, we used DCT to concentrate the information of PSSM into a few coefficients. For a given input matrix $Mat\in {ℜ}^{m\times n}$, DCT is defined as:

(2) $$DCT\left(i,j\right)=a_{i}a_{j}\overset{M-1}{\underset{m=0}{\sum }}\overset{N-1}{\underset{n=0}{\sum }}Mat\left(m,n\right)\cos\frac{\pi \left(2m+1\right)i}{2M}\times \cos\frac{\pi \left(2n+1\right)j}{2N},\;0\leq i\leq M,\;\;\;0\leq j\leq N,$$

where

(3) $$a_{i}=\{\begin{array}{l} \frac{1}{\sqrt{M}},\;\;i=0\\ \sqrt{\frac{2}{M}},\;\;1\leq i\leq M-1 \end{array}\;a_{j}=\{\begin{array}{l} \frac{1}{\sqrt{N}},\;\;j=0\\ \sqrt{\frac{2}{N}},\;\;1\leq j\leq N-1 \end{array}$$

The compressed PSSM feature of the residue was obtained by using DCT on the PSSM feature matrix. Most of the information after PSSM-DCT was concentrated in the low-frequency part of the compressed PSSM. The first *r* rows of the compressed PSSM were reserved as the PSSM-DCT feature, and the dimensions of the PSSM-DCT feature were *r ×* 20.

#### 2.2.3. Discrete Wavelet Transform

**Figure 3:**
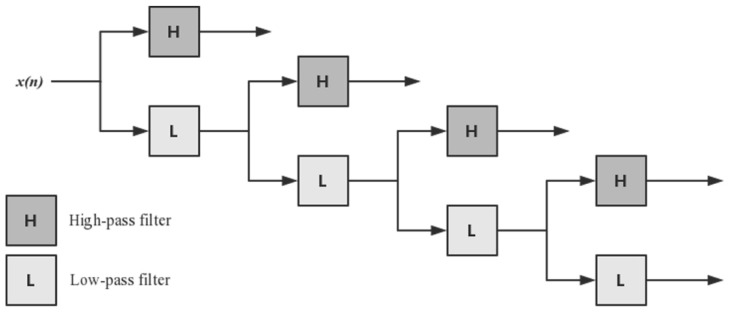
Four-level discrete wavelet transform (DWT).

Discrete Wavelet Transform (DWT) [49] can decompose discrete sequences into high- and low-frequency coefficients. Four-level DWT [50] was applied to acquire the first five discrete cosine coefficients, the standard deviation, mean, and maximum and minimum values of different scales, as shown in Figure 3. The PSSM-DWT feature of the residue was obtained from the PSSM feature via four-level DWT, and the PSSM-DWT feature had 1040 dimensions.

#### 2.2.4. Predicting Solvent Accessibility

Solvent accessibility [52] is related to the spatial arrangement and packing of residues during the protein folding process, which is an effective feature for protein–ligand binding site prediction [8,33,34]. We used the solvent accessibility prediction of proteins by nearest neighbor method (Sann) to obtain the PSA feature of residues [55], and the PSA feature had three dimensions.

### 2.3. SMOTE Over-Sampling

As a common method for tackling unbalanced data, SMOTE over-samples the minority class by synthesizing new samples, under-samples the majority class, and provides better classifier performance within the receiver operating characteristic (ROC) space [45,56]. A balanced sample set is generated from the unbalanced sample set through feature extraction by SMOTE. After a series of tests, a new sample set with better results was constructed with the same positive and negative sample number: 19,000.

### 2.4. Extreme Gradient Boosting Algorithm

Extreme Gradient Boosting (XGBoost) algorithm [46] is an improvement on the Gradient Boosting algorithm [57] by Chen et al. and is characterized by fast calculation and high prediction accuracy. XGBoost is widely used by data scientists in multiple applications and has provided advanced results [58,59]. The training set after feature extraction and SMOTE $x_{i}$ ($x_{i}=\left\{x_{1},x_{2},\ldots ,x_{m}\right\},i=1,2,\ldots ,n\;\;$) was input into the K additive functions of XGBoost to build the model. The prediction result of the independent test set $y_{i}$ ($y_{i}=\left\{0,1\right\},i=1,2,\ldots ,s$, where 0 represents non-binding residues and 1 represents binding residues) was output as follows:

(4) $${\hat{y}}_{i}=\overset{K}{\underset{k=1}{\sum }}f_{k}\left(x_{i}\right),\;\;\;\;f_{k}\in F$$

where $f_{k}$ is each independent tree function with leaf weights and *F* is the tree ensemble containing each function of the tree. XGBoost avoids large models with the following regularized objective formula:

(5) $$ℒ\left(\phi \right)=\underset{i}{\sum }l\left({\hat{y}}_{i},y_{i}\right)+\underset{k}{\sum }\Omega \left(f_{k}\right)$$

where *l* is a differentiable convex loss function that measures the closeness of the prediction ${\hat{y}}_{i}$ and the target $y_{i}$, and Ω is a regular term that penalizes model complexity by greedily adding $f_{t}$ to improve the tree ensemble model. The regular term avoids overfitting by penalizing leaf weights, and the Ω penalty function is as follows:

(6) $$\Omega \left(f\right)=\gamma T+\frac{1}{2}\lambda {\left\|\omega \right\|}^{2}$$

where *T* is the number of leaves, ω is the leaf weights, and the regularization coefficients γ and λ are constants. The traditional GBDT only uses the first-order information of the loss function, whereas the second-order Taylor expansion was introduced into the loss function of XGBoost to optimize the function rapidly [60]. The simplified objective function of step *t* is:

(7) $${\tilde{ℒ}}^{\left(t\right)}=\overset{n}{\underset{i=1}{\sum }}\left[g_{i}f_{t}\left(x_{i}\right)+\frac{1}{2}h_{i}f_{t}^{2}\left(x_{i}\right)\right]+\Omega \left(f_{t}\right)$$

where $g_{i}=\partial_{{\hat{y}}^{\left(t-1\right)}}l\left(y_{i},{\hat{y}}^{\left(t-1\right)}\right)$ and $h_{i}=\partial_{{\hat{y}}^{\left(t-1\right)}}^{2}l\left(y_{i},{\hat{y}}^{\left(t-1\right)}\right)$ represent the first- and second-order gradient statistics of the loss function, respectively. $I_{j}=\left\{i|q\left(x_{i}=j\right)\right\}$ is defined as a sample set of leaf *j*, simplified Equation (7) is:

(8) $${\tilde{ℒ}}^{\left(t\right)}=\overset{n}{\underset{i=1}{\sum }}\left[g_{i}f_{t}\left(x_{i}\right)+\frac{1}{2}h_{i}f_{t}^{2}\left(x_{i}\right)\right]+\gamma T+\frac{1}{2}\lambda \overset{T}{\underset{j=1}{\sum }}\omega_{j}^{2}\;=\overset{T}{\underset{j=1}{\sum }}\left[\left(\underset{i\in I_{j}}{\sum }g_{i}\right)\omega_{j}+\frac{1}{2}\left(\underset{i\in I_{j}}{\sum }h_{i}+\lambda \right)\omega_{j}^{2}\right]+\gamma T$$

The optimal weight $\omega_{j}^{*}$ of leaf *j* and the corresponding objective function value are calculated by:

(9) $$\omega_{j}^{*}=-\frac{\sum_{i\in I_{j}}g_{i}}{\sum_{i\in I_{j}}h_{i}+\lambda }$$

(10) $$\;\;{\tilde{ℒ}}^{\left(t\right)}\left(q\right)=-\frac{1}{2}\overset{T}{\underset{j=1}{\sum }}\frac{{\left(\sum_{i\in I_{j}}g_{i}\right)}^{2}}{\sum_{i\in I_{j}}h_{i}+\lambda }+\gamma T$$

The above equation provides the best split of the node. Supposing $I_{L}$ and $I_{R}$ are the left and right split nodes of the sample set *I* of the leaf, $\;I=I_{L}\cup I_{R}$, respectively, the loss reduction after splitting is expressed as:

(11) $${ℒ}_{split}=\frac{1}{2}\left[\frac{{\left(\sum_{i\in I_{L}}g_{i}\right)}^{2}}{\sum_{i\in I_{L}}h_{i}+\lambda }+\frac{{\left(\sum_{i\in I_{R}}g_{i}\right)}^{2}}{\sum_{i\in I_{R}}h_{i}+\lambda }-\frac{{\left(\sum_{i\in I}g_{i}\right)}^{2}}{\sum_{i\in I}h_{i}+\lambda }\right]-\gamma$$

To prevent overfitting, XGBoost uses shrinkage and column (feature) subsampling techniques, as well as the regularized objective [57].

## 3. Results

The performance of classification was evaluated on the specificity (SP), sensitivity (SN), accuracy (ACC), and Matthews correlation coefficient (MCC). The overall prediction quality of a binary model was evaluated using the area under the receiver operating characteristic curve (AUC). The formulas used to determine SN, SP, ACC, and MCC are, respectively, as follows:

(12) $$\mathrm{SP}=\frac{TN}{TN+FP}$$

(13) $$\;\mathrm{SN}=\frac{TP}{TP+FN}$$

(14) $$\;\mathrm{ACC}=\frac{TP+TN}{TP+FP+TN+FN}$$

(15) $$\;\mathrm{MCC}=\frac{TP\times TN-FP\times FN}{\sqrt{\left(TP+FN\right)\times \left(TN+FP\right)\times \left(TP+FP\right)\times \left(TN+FN\right)}}$$

where *TP*, *FP*, *TN,* and *FN* represent true positive, false positive, true negative, and false negative, respectively.

### 3.1. Parameter Selection

**Figure 4:**
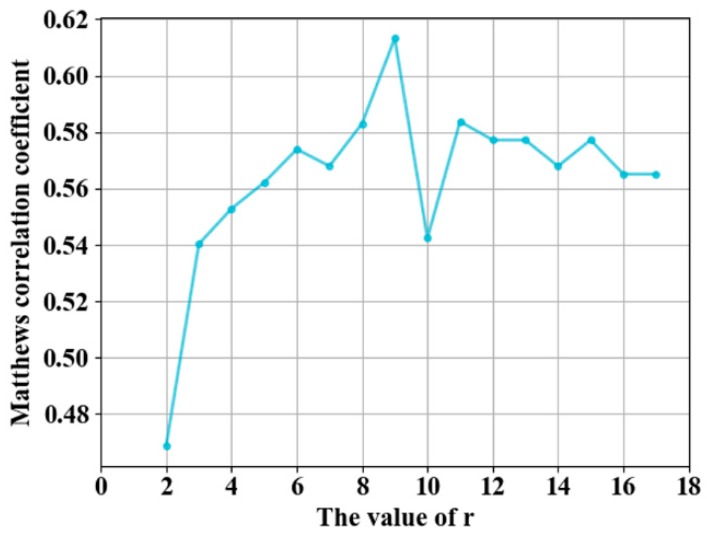
Adjustment of parameters *r.*

ACC is insufficient for performance evaluation in unbalanced learning [7,8], MCC is suitable for quality assessment in sequence-based predictions [3], and AUC is usually used to assess the overall prediction quality of models. The value of MCC changes with the threshold, whereas the AUC value is not affected by the threshold value. We evaluated the prediction performance using MCC and AUC, and the threshold of the probability value was selected by maximizing the value of MCC. The value of MCC was used to select the first *r* rows of the PSSM-DCT matrix as feature on the guanosine triphosphate (GTP) training and independent test sets. PSSM-DCT obtained the optimal value of MCC when *r* was 9, as shown in Figure 4, and the dimensions of the PSSM-DCT feature were 9 × 20 = 180.

**Figure 5:**
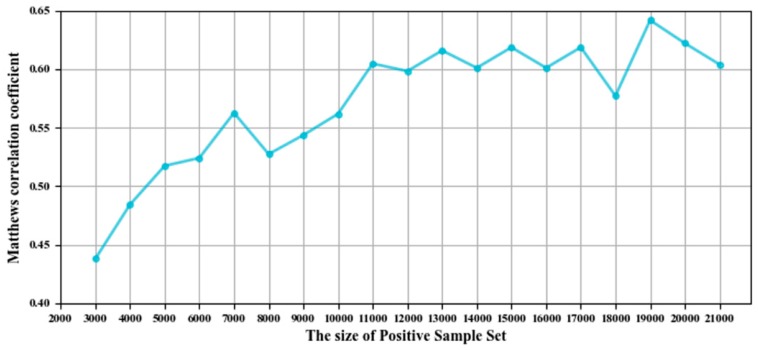
The values of the Matthews correlation coefficient (MCC) corresponding to the number of samples after SMOTE.

The size of the positive and negative sample sets after SMOTE is usually an integer multiple of the positive sample size in the original dataset, and the prediction quality may be affected by the amplification ratio of the positive sample sets. In this study, a fixed-size positive and negative sample set was generated by SMOTE to improve the prediction quality, and the optimal sample number was selected according to the value of MCC on the GTP training and independent test sets. The best value of MCC was obtained when the number of positive and negative samples was 19,000, as shown in Figure 5.

The parameters of XGBoost were adjusted with five-fold cross-validation and a grid search method on the GTP training set.

### 3.2. Method Selection

**Figure 6:**
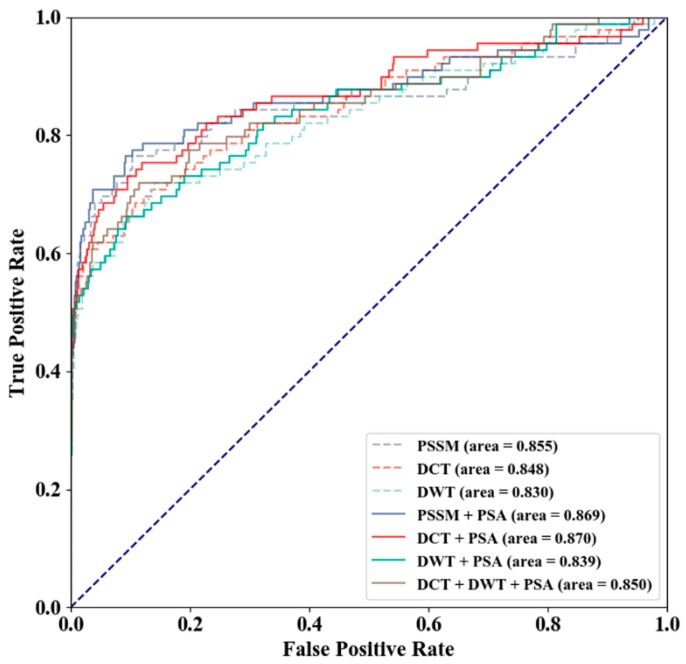
Receiver Operating Characteristic Curve (ROC) of Different Feature Combinations.

Different feature combinations of PSSM, PSSM-DCT, PSSM-DWT, and PSA were used to evaluate the prediction performance using the GTP training and independent test sets, PSSM-DCT + PSA produced the optimal MCC and AUC values (Table 2), and receiver operating characteristic curve (ROC) of different feature combinations is shown in Figure 6. As shown in Table 2, PSSM performed better in terms of AUC than PSSM-DCT and PSSM-DWT, and PSA (3-D) improved PSSM (340-D), PSSM-DCT (180-D), and PSSM-DWT (1040-D) by 0.14, 0.22 and 0.09, respectively. The relationship between the increase in AUC and the feature dimensions indicated that the prediction quality using PSA improved more for features with fewer dimensions (PSSM and PSSM-DCT). PSSM + PSA and PSSM-DCT + PSA performed almost the same in terms of AUC, and we tended to improve prediction quality by over-sampling in the comparison of feature combinations. The prediction qualities of PSSM and PSSM + PSA were more dependent on threshold moving, and the difference in MCC between the default threshold (0.500) and the maximum MCC threshold demonstrated the effect of threshold moving.

**Figure 7:**
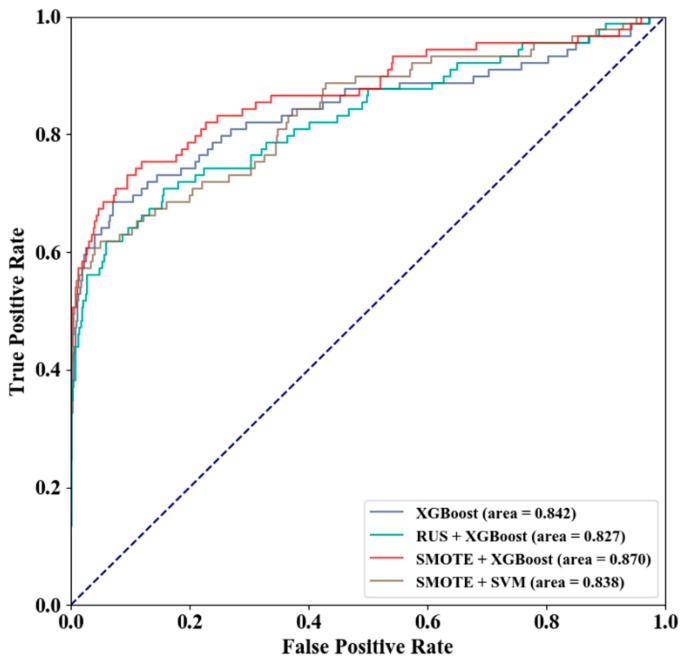
ROC of Different Sampling and Classification Algorithms.

Three sampling schemes were used on the GTP training set to obtain three different training sets, including the entire GTP training set, the training set after random under-sampling (RUS), and the training set after SMOTE over-sampling. On the GTP independent test set, the prediction qualities of the models constructed by the three training sets are shown in Table 3, and receiver operating characteristic curve (ROC) of different sampling and classification algorithms is shown in Figure 7. SMOTE + XGBoost achieved the best prediction quality, performing better than SMOTE + SVM.

### 3.3. Results of Training Sets

The performance of SXGBsite was evaluated using five-fold cross-validation on the training sets. The results with the threshold of 0.5 and the maximized the MCC value are listed in Table 4. The five-fold cross-validation results are basically consistent with the maximized MCC threshold results of TargetS and EC-RUS. Regardless of the impact of the threshold, the results in Table 4 show the different characteristics of the two schemes for the class imbalance problem by comparing the default threshold (0.500) results of SXGBsite and EC-RUS, which use the same features. The RUS + ensemble classifiers scheme was more sensitive to positive samples and had information loss for negative samples. The SMOTE + XGBoost scheme reduced the information loss, the positive samples in the training set were mostly synthesized, and the sensitivity to positive samples was lower.

### 3.4. Comparison with Existing Methods

In terms of the independent test sets of the five nucleotides, SXGBsite is compared with TargetS, SVMPred, NsitePred, EC-RUS, and the alignment-based baseline predictor in Table 5. The results of TargetS, SVMPred, NsitePred, and EC-RUS are the threshold of maximizing the MCC value. In terms of the ATP, ADP, AMP, GDP, and GTP independent test sets, the metrics of the best prediction quality refer to the AUC of TargetS and the MCC of EC-RUS. The differences between SXGBsite and TargetS for the AUC are 0.018 (0.880 to 0.898), 0.011 (0.885 to 0.896), 0.007 (0.823 to 0.830), 0.002 (0.894 to 0.896), and 0.015 (0.870 over 0.855), respectively, and the differences between SXGBsite and EC-RUS for the MCC are 0.043 (0.463 to 0.506), 0.023 (0.488 to 0.511), 0.065 (0.328 to 0.393), 0.003 (0.576 to 0.579), and 0.009 (0.650 over 0.641), respectively. The difference between SXGBsite and the best prediction quality is small for the AUC and relatively large for the MCC.

On the independent test sets of the five metal ions, SXGBsite is compared with TargetS, FunFOLD, CHED, EC-RUS, and the alignment-based baseline predictor in Table 6. The results of TargetS, FunFOLD, CHED, and EC-RUS are the threshold of maximizing the MCC value. In terms of the independent test sets of ${\mathrm{Ca}}^{2+}$, ${\mathrm{Mg}}^{2+}$, ${\mathrm{Mn}}^{2+}$, ${\mathrm{Fe}}^{3+}$, and ${\mathrm{Zn}}^{2+}$, the differences between SXGBsite and the best prediction quality for the AUC are 0.021 (0.758 to 0.779), 0.001 (0.779 to 0.780), 0.032 (0.856 to 0.888), 0.054 (0.891 to 0.945), and 0.052 (0.906 to 0.958), respectively, and the differences between SXGBsite and the best prediction quality for the MCC are 0.046 (0.197 to 0.243), 0.026 (0.291 to 0.317), 0.067 (0.382 to 0.449), 0.094 (0.396 to 0.490), and 0.137 (0.390 to 0.527), respectively. SXGBsite showed good prediction performance on the ${\mathrm{Mg}}^{2+}$ independent test set, and the reasons for the unsatisfactory performance on the metal ion independent test sets may be as follows: (1) TargetS uses the ligand-specific binding propensity feature to improve the prediction quality, and the features used in this study did not perform well for predicting metal ion binding residues; and (2) the volume of metal ions is smaller than that of nucleotides, which means that there are fewer binding residues (positive samples), and the lack of positive samples affected the prediction quality of the model.

Compared with TargetS, MetaDBSite, DNABR, EC-RUS, and the alignment-based baseline predictor on the DNA independent test set (Table 7), SXGBsite achieved an MCC value lower than those of TargetS and EC-RUS, and an inferior AUC value to TargetS.

Compared with TargetS, HemeBind, EC-RUS, and the alignment-based baseline predictor on the Heme independent test set (Table 8), SXGBsite achieved inferior MCC and AUC values to EC-RUS.

The prediction performance of SXGBsite was similar to those of the best two methods, TargetS and EC-RUS, on the independent test sets of the five nucleotides, ${\mathrm{Mg}}^{2+}$, DNA, and Heme. Both TargetS and EC-RUS are serial combinations of under-sampling and ensemble classifiers, which requires long calculation times. SXGBsite is a method of over-sampling and a single XGBoost classifier to quickly build high quality prediction models.

### 3.5. Running Time Comparison

The running time comparison of SXGBsite, EC-RUS (SVM), and EC-RUS (WSRC) on the independent test sets is provided in Table 9, and the benchmark in this study is the EC-RUC (SVM) running time. EC-RUS is a sequence-based method that was proposed by Ding et al., and its prediction quality was excellent. Ding et al. selected 19 sub-classifiers in the ensemble classifier, compared the results of ensemble SVMs and ensemble WSRCs, and concluded that ensemble WSRCs are more time-consuming than ensemble SVMs. Both SXGBsite and EC-RUS used the feature of PSSM-DCT + PSA, and the prediction model was built by SMOTE + XGBoost and RUS + ensemble classifiers, respectively. Due to having the same features, the results in Table 9 also show the running time comparison of SMOTE + XGBoost and RUS + ensemble classifiers, which means that two schemes for the class imbalance problem.

### 3.6. Comparison with Existing Methods on the PDNA-41 Independent Test Set

Different from the previous protein–DNA binding site dataset, PDNA-543 (9549 binding residues and 134,995 non-binding residues) and PDNA-41 (734 binding residues and 14,021 non-binding residues) are datasets constructed by Hu et al. [61]. SXGBsite constructed the prediction model by the PDNA-543 training set, obtained prediction results on the PDNA-41 independent test set, and the comparison of SXGBsite with BindN [37], ProteDNA [62], BindN+ [63], MetaDBSite [32], DP-Bind [39], DNABind [64], TargetDNA [61], and EC-RUS(DNA) [44] is provided in Table 10. SXGBsite achieved the best MCC (0.272) under *Sen* ≈ *Spec,* and achieved MCC after EC-RUS(DNA) and TargetDNA under *FPR* ≈ 5% (*FPR* = 1 - SP). The best MCC (0.279) of SXGBsite is achieved under *FPR* ≈ 10%.

### 3.7. Case Study

**Figure 8:**
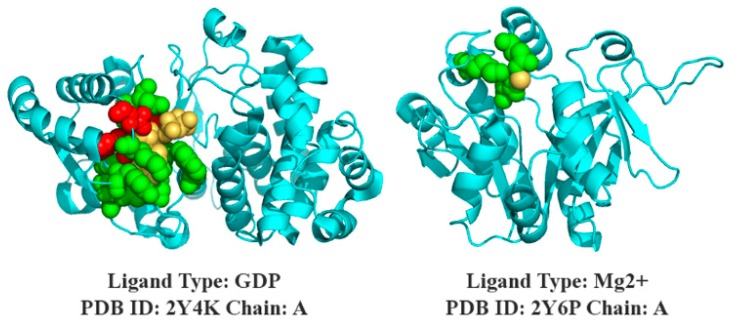
Prediction of SXGBsite. The cyan indicates the helix, the folding and ring structure of the protein sequence, and the yellow indicates the ligand; and true and false predictions are indicated in green and red, respectively.

The prediction results of SXGBsite are shown in the 3D models in Figure 8, and the protein–ligand complexes of 2Y4K-A and 2Y6P-A belong to the independent test sets of GDP and ${\mathrm{Mg}}^{2+}$, respectively.

## 4. Discussion

Many excellent computational methods are available in the field of protein–ligand binding site prediction; however, prediction efficiency can still be improved [8]. As the actual acquired protein–ligand binding site data show many fewer binding sites than non-binding sites, we selected unbalanced datasets of 12 different ligand types constructed by Yu et al. as the benchmark datasets. The adverse effects of unbalanced data on predictions are usually mitigated by over- or under-sampling methods, which are widely applied, and ensemble classifiers are often used together to overcome the loss of information caused by under-sampling. Both TargetS and EC-RUS performed well on the independent test sets built by Yu et al. by applying the scheme of under-sampling and ensemble classifiers. Although the loss of information by multiple under-sampling can be reduced by ensemble classifiers, serial combinations of multiple machine learning algorithms and high-dimensional features increase the computation time.

SXGBsite uses the features of PSSM-DCT + PSA and XGBoost with SMOTE to build prediction models, and Extreme Gradient Boosting algorithm developed by Chen et al. [46] was applied to solve overfitting and large sample sets caused by over-sampling. XGBoost’s regularization technology overcomes the overfitting problem, and parallel computing can be used to quickly construct prediction models with large sample sets, which constitute the basis of SXGBsite. The threshold moving was used in this study to obtain the best MCC for comparison with other existing methods. The use of both threshold moving and sampling methods complicated the interpretation of the results, and the AUC measure without threshold change was used to better evaluate the prediction quality difference between SMOTE + XGBoost and RUS + ensemble classifiers. On the independent test sets of five nucleotides, ${\mathrm{Mg}}^{2+}$, DNA, and Heme, the difference between the AUC of SXGBsite and the best AUC was within 0.020. Considering the decrease in the running time, we think that the difference in AUC is acceptable. On the independent test sets of 12 ligands, the new method proposed here produced a higher prediction quality with a shorter computation time using the two features and a single classifier, and produced similar results to the best-performing TargetS and EC-RUS on 8 of the 12 independent test sets.

## 5. Conclusions

This paper proposes a new computational method, SXGBsite. Sequence information was used for the protein–ligand binding site prediction, and features extracted by PSSM-DCT+PSA and XGBoost with SMOTE were used to construct the prediction model. On the independent test sets of 12 different ligands, SXGBsite performed similarly to the best methods on the datasets with less computation time, which could be a complement of biological experiments as well as cost reductions. The features we used did not perform well on the metal ion datasets, and adding features with better prediction performance is the next step of the study.

## Figures and Tables

**Table 1:** Composition of datasets for the 12 different ligands [7].

Ligand Category	Ligand Type	Training Dataset		Independent Test Dataset		Total No. Sequences
		No. Sequences	(numP,numN)	No. Sequences	(numP,numN)	
Nucleotide	ATP	221	(3021,72334)	50	(647,16639)	271
	ADP	296	(3833,98740)	47	(686,20327)	343
	AMP	145	(1603,44401)	33	(392,10355)	178
	GDP	82	(1101,26244)	14	(194,4180)	96
	GTP	54	(745,21205)	7	(89,1868)	61
Metal Ion	${\mathrm{Ca}}^{2+}$	965	(4914,287801)	165	(785,53779)	1130
	${\mathrm{Zn}}^{2+}$	1168	(4705,315235)	176	(744,47851)	1344
	${\mathrm{Mg}}^{2+}$	1138	(3860,350716)	217	(852,72002)	1355
	${\mathrm{Mn}}^{2+}$	335	(1496,112312)	58	(237,17484)	393
	${\mathrm{Fe}}^{3+}$	173	(818,50453)	26	(120,9092)	199
	DNA	335	(6461,71320)	52	(973,16225)	387
	HEME	206	(4380,49768)	27	(580,8630)	233

Note: numP, positive (binding residues) sample numbers; numN, negative (non-binding residues) sample numbers; ATP, adenosine triphosphate; ADP, adenosine diphosphate; AMP, adenosine monophosphate; GDP, guanosine diphosphate; GTP, guanosine triphosphate.

**Table 2:** Comparison of different feature combinations on the GTP independent test set (average of 10 replicate experiments in SXGBsite with adjusted parameters).

Feature	Threshold	SN (%)	SP (%)	ACC (%)	MCC	AUC
PSSM	0.500	34.8	99.7	96.8	0.536	0.855
	0.139	46.1	99.5	97.0	0.596	0.855
PSSM-DCT	0.500	43.8	99.7	97.1	0.605	0.848
	0.612	42.7	99.8	97.2	0.611	0.848
PSSM-DWT	0.500	41.6	99.7	97.0	0.586	0.830
	0.458	43.8	99.7	97.1	0.605	0.830
PSSM + PSA	0.500	37.1	99.9	97.0	0.581	0.869
	0.109	52.8	99.4	97.2	0.636	0.869
PSSM-DCT + PSA	0.500	49.4	99.6	97.3	0.642	0.870
	0.421	50.6	99.6	97.4	0.650	0.870
PSSM-DWT + PSA	0.500	46.1	99.7	97.3	0.630	0.839
	0.370	49.4	99.6	97.3	0.642	0.839
PSSM-DCT + PSSM-DWT + PSA	0.500	44.9	99.6	97.1	0.607	0.850
	0.545	44.9	99.8	97.3	0.629	0.850

Note: ACC, accuracy; MCC, Matthews correlation coefficient; AUC, the area under the receiver operating characteristic curve.

**Table 3:** Comparison of different sampling and classification algorithms on the GTP independent test set (average of 10 replicate experiments in XGBoost with adjusted parameters).

Scheme	Threshold	SN (%)	SP (%)	ACC (%)	MCC	AUC
XGBoost	0.500	30.3	99.8	96.7	0.512	0.842
	0.153	37.1	99.7	96.9	0.556	0.842
RUS + XGBoost	0.500	68.5	84.5	83.8	0.288	0.827
	0.914	51.7	97.9	95.8	0.504	0.827
SMOTE + XGBoost	0.500	49.4	99.6	97.3	0.642	0.870
	0.421	50.6	99.6	97.4	0.650	0.870
SMOTE + SVM	0.500	51.7	99.3	97.1	0.616	0.838
	0.714	49.4	99.5	97.2	0.628	0.838

**Table 4:** Performance of SXGBsite (average of 10 replicate experiments) on the training sets after five-fold cross-validation.

Ligand	Predictor	Threshold	SN (%)	SP (%)	ACC (%)	MCC	AUC
ATP	TargetS ^1^	0.500	48.4	98.2	96.2	0.492	0.887
	EC-RUS ^2^	0.500	84.1	84.9	84.9	0.347	0.912
		0.814	58.6	97.9	96.4	0.537	0.912
	SXGBsite	0.500	53.4	96.3	94.6	0.413	0.886
		0.775	40.3	98.6	96.4	0.448	0.886
ADP	TargetS ^1^	0.500	56.1	98.8	97.2	0.591	0.907
	EC-RUS ^2^	0.500	87.8	87.7	87.7	0.395	0.939
		0.852	62.2	98.6	97.3	0.610	0.939
	SXGBsite	0.500	61.6	96.2	94.9	0.459	0.907
		0.832	46.4	98.9	97.0	0.521	0.907
AMP	TargetS ^1^	0.500	38.0	98.2	96.0	0.386	0.856
	EC-RUS ^2^	0.500	81.5	79.7	79.8	0.263	0.888
		0.835	46.7	98.3	96.6	0.460	0.888
	SXGBsite	0.500	37.0	97.8	95.8	0.347	0.851
		0.636	32.3	98.6	96.4	0.366	0.851
GDP	TargetS ^1^	0.430	63.9	98.7	97.2	0.644	0.908
	EC-RUS ^2^	0.500	86.1	89.8	89.7	0.435	0.937
		0.816	67.2	98.9	97.6	0.676	0.937
	SXGBsite	0.500	59.4	99.3	97.7	0.664	0.930
		0.653	57.0	99.5	97.9	0.678	0.930
GTP	TargetS ^1^	0.500	48.0	98.7	96.9	0.506	0.858
	EC-RUS ^2^	0.500	79.5	85.7	85.5	0.309	0.896
		0.842	49.5	99.2	97.6	0.562	0.896
	SXGBsite	0.500	42.4	99.4	97.6	0.540	0.883
		0.685	40.7	99.7	97.8	0.572	0.883
${\mathrm{Ca}}^{2+}$	TargetS ^1^	0.690	19.2	99.7	98.4	0.320	0.784
	EC-RUS ^2^	0.500	73.9	73.8	73.8	0.118	0.812
		0.861	14.7	99.7	98.6	0.220	0.812
	SXGBsite	0.500	32.8	95.0	94.2	0.135	0.757
		0.818	16.3	99.1	98.1	0.167	0.757
${\mathrm{Mg}}^{2+}$	TargetS ^1^	0.810	26.4	99.8	99.0	0.383	0.798
	EC-RUS ^2^	0.500	73.8	79.4	79.3	0.125	0.839
		0.864	25.8	99.8	99.1	0.354	0.839
	SXGBsite	0.500	46.1	95.9	95.5	0.196	0.819
		0.926	26.3	99.7	99.0	0.326	0.819
${\mathrm{Mn}}^{2+}$	TargetS ^1^	0.740	40.8	99.5	98.7	0.445	0.901
	EC-RUS ^2^	0.500	83.4	86.6	86.6	0.201	0.921
		0.841	31.0	99.6	98.9	0.358	0.921
	SXGBsite	0.500	45.0	98.3	97.7	0.297	0.888
		0.759	36.1	99.1	98.5	0.329	0.888
${\mathrm{Fe}}^{3+}$	TargetS ^1^	0.810	51.8	99.6	98.8	0.592	0.922
	EC-RUS ^2^	0.500	87.1	90.1	90.0	0.278	0.940
		0.809	53.1	99.2	98.6	0.489	0.940
	SXGBsite	0.500	48.2	99.1	98.5	0.440	0.913
		0.496	50.1	99.1	98.5	0.454	0.913
${\mathrm{Zn}}^{2+}$	TargetS ^1^	0.830	50.0	99.6	98.9	0.557	0.938
	EC-RUS ^2^	0.500	88.7	90.8	90.8	0.279	0.958
		0.860	45.6	99.3	98.7	0.440	0.958
	SXGBsite	0.500	59.7	96.5	96.1	0.299	0.892
		0.894	38.5	99.2	98.5	0.363	0.892
DNA	TargetS ^1^	0.490	41.7	94.5	89.9	0.362	0.824
	EC-RUS ^2^	0.500	81.9	71.8	72.3	0.259	0.852
		0.763	48.7	95.1	92.6	0.378	0.852
	SXGBsite	0.500	41.0	92.3	89.6	0.255	0.827
		0.420	49.8	89.2	87.2	0.270	0.827
HEME	TargetS ^1^	0.650	50.5	98.3	94.4	0.579	0.887
	EC-RUS ^2^	0.500	85.0	83.6	83.7	0.416	0.922
		0.846	60.3	97.5	95.1	0.591	0.922
	SXGBsite	0.500	59.3	96.2	93.8	0.520	0.900
		0.805	45.3	98.9	95.4	0.555	0.900

^1^ Results excerpted from Yu et al. [7]. ^2^ Results excerpted from Ding et al. [8].

**Table 5:** SXGBsite (average of 10 replicate experiments) compared with the existing methods on five nucleotide independent test sets.

Ligand	Predictor	SN (%)	SP (%)	ACC (%)	MCC	AUC
ATP	TargetS ^1^	50.1	98.3	96.5	0.502	0.898
	NsitePred ^1^	50.8	97.3	95.5	0.439	-
	SVMPred ^1^	47.3	96.7	94.9	0.387	0.877
	alignment-based ^1^	30.6	97.0	94.5	0.265	-
	EC-RUS ^2^	45.4	98.8	96.8	0.506	0.871
	SXGBsite (T = 0.500)	54.6	95.7	94.2	0.397	0.880
	SXGBsite (T = 0.718)	43.7	98.5	96.5	0.463	0.880
ADP	TargetS ^1^	46.9	98.9	97.2	0.507	0.896
	NsitePred ^1^	46.2	97.6	96.0	0.419	-
	SVMPred ^1^	46.1	97.2	95.5	0.382	0.875
	alignment-based ^1^	31.8	97.4	95.1	0.284	-
	EC-RUS ^2^	44.4	99.2	97.6	0.511	0.872
	SXGBsite (T = 0.500)	53.1	96.9	95.6	0.399	0.885
	SXGBsite (T = 0.844)	37.3	99.5	97.7	0.488	0.885
AMP	TargetS ^1^	34.2	98.2	95.9	0.359	0.830
	NsitePred ^1^	33.9	97.6	95.3	0.321	-
	SVMPred ^1^	32.1	96.4	94.1	0.255	0.798
	alignment-based ^1^	19.6	97.3	94.5	0.178	-
	EC-RUS ^2^	24.9	99.5	97.0	0.393	0.815
	SXGBsite (T = 0.500)	36.0	97.5	95.4	0.325	0.823
	SXGBsite (T = 0.486)	37.1	97.4	95.3	0.328	0.823
GDP	TargetS ^1^	56.2	98.1	96.2	0.550	0.896
	NsitePred ^1^	55.7	97.9	96.1	0.536	-
	SVMPred ^1^	49.5	97.6	95.4	0.466	0.870
	alignment-based ^1^	41.2	97.8	95.3	0.415	-
	EC-RUS ^2^	36.6	99.9	97.1	0.579	0.872
	SXGBsite (T = 0.500)	46.4	99.0	96.7	0.551	0.894
	SXGBsite (T = 0.687)	40.2	99.7	97.1	0.576	0.894
GTP	TargetS ^1^	57.3	98.8	96.9	0.617	0.855
	NsitePred ^1^	58.4	95.7	94.0	0.448	-
	SVMPred ^1^	48.3	91.7	89.7	0.276	0.821
	alignment-based ^1^	52.8	97.9	95.9	0.516	-
	EC-RUS ^2^	61.8	98.7	97.0	0.641	0.861
	SXGBsite (T = 0.500)	49.4	99.6	97.3	0.642	0.870
	SXGBsite (T = 0.421)	50.6	99.6	97.4	0.650	0.870

^1^ Results excerpted from Yu et al. [7]. ^2^ Results excerpted from Ding et al. [8]. - denotes unavailable.

**Table 6:** SXGBsite (average of 10 replicate experiments) compared with the existing methods on the five metal ion independent test sets.

Ligand	Predictor	SN (%)	SP (%)	ACC (%)	MCC	AUC
${\mathrm{Ca}}^{2+}$	TargetS ^1^	13.8	99.8	98.8	0.243	0.767
	FunFOLD ^1^	12.2	99.6	98.1	0.196	-
	CHED ^1^	18.7	98.2	97.1	0.142	-
	alignment-based ^1^	20.3	98.6	97.5	0.175	-
	EC-RUS ^2^	17.3	99.6	98.7	0.225	0.779
	SXGBsite (T = 0.500)	32.6	95.6	94.9	0.139	0.758
	SXGBsite (T = 0.832)	13.3	99.7	98.7	0.197	0.758
${\mathrm{Mg}}^{2+}$	TargetS ^1^	18.3	99.8	98.8	0.294	0.706
	FunFOLD ^1^	22.0	99.1	98.3	0.215	-
	CHED ^1^	14.6	98.3	97.3	0.103	-
	alignment-based ^1^	14.1	99.2	98.2	0.147	-
	EC-RUS ^2^	20.1	99.8	99.1	0.317	0.780
	SXGBsite (T = 0.500)	41.0	96.3	95.8	0.177	0.779
	SXGBsite (T = 0.917)	19.8	99.8	99.1	0.291	0.779
${\mathrm{Mn}}^{2+}$	TargetS ^1^	40.1	99.5	98.7	0.449	0.888
	FunFOLD ^1^	23.3	99.8	98.7	0.376	-
	CHED ^1^	35.0	98.1	97.3	0.253	-
	alignment-based ^1^	26.6	99.0	98.0	0.257	-
	EC-RUS ^2^	35.8	99.6	98.9	0.403	0.888
	SXGBsite (T = 0.500)	44.3	98.3	97.7	0.299	0.856
	SXGBsite (T = 0.797)	34.2	99.5	98.8	0.382	0.856
${\mathrm{Fe}}^{3+}$	TargetS ^1^	48.3	99.3	98.7	0.479	0.945
	FunFOLD ^1^	47.2	99.1	98.4	0.432	-
	CHED ^1^	49.2	97.0	96.3	0.279	-
	alignment-based ^1^	30.0	99.2	98.3	0.300	-
	EC-RUS ^2^	44.3	99.6	99.0	0.490	0.936
	SXGBsite (T = 0.500)	42.5	99.0	98.3	0.361	0.891
	SXGBsite (T = 0.670)	38.7	99.4	98.7	0.396	0.891
${\mathrm{Zn}}^{2+}$	TargetS ^1^	46.4	99.5	98.7	0.527	0.936
	FunFOLD ^1^	36.5	99.5	98.6	0.436	-
	CHED ^1^	37.9	98.0	97.1	0.280	-
	alignment-based ^1^	29.7	99.0	98.0	0.297	-
	EC-RUS ^2^	48.9	99.2	98.6	0.437	0.958
	SXGBsite (T = 0.500)	62.4	96.7	96.3	0.323	0.906
	SXGBsite (T = 0.833)	41.0	99.2	98.6	0.390	0.906

^1^ Results excerpted from Yu et al. [7]. ^2^ Results excerpted from Ding et al. [8]. - denotes unavailable.

**Table 7:** SXGBsite (average of 10 replicate experiments) compared with the existing methods on the DNA independent test set.

Ligand	Predictor	SN (%)	SP (%)	ACC (%)	MCC	AUC
DNA	TargetS ^1^	41.3	96.5	93.3	0.377	0.836
	MetaDBSite ^1^	58.0	76.4	75.2	0.192	-
	DNABR ^1^	40.7	87.3	84.6	0.185	-
	alignment-based ^1^	26.6	94.3	90.5	0.190	-
	EC-RUS ^2^	31.5	97.8	95.2	0.319	0.814
	SXGBsite (T = 0.500)	36.5	95.1	92.8	0.256	0.826
	SXGBsite (T = 0.408)	46.2	92.8	91.0	0.269	0.826

^1^ Results excerpted from Yu et al. [7]. ^2^ Results excerpted from Ding et al. [8]. - denotes unavailable.

**Table 8:** SXGBsite (average of 10 replicate experiments) compared with the existing methods on the HEME independent test set.

Ligand	Predictor	SN (%)	SP (%)	ACC (%)	MCC	AUC
HEME	TargetS (T = 0.650) ^1^	49.8	99.0	95.9	0.598	0.907
	TargetS(T = 0.180) ^1^	69.3	90.4	89.1	0.426	0.907
	HemeBind ^1^	86.2	90.7	90.6	0.537	-
	alignment-based ^1^	51.4	97.3	94.4	0.507	-
	EC-RUS (T = 0.500) ^2^	83.5	87.5	87.3	0.453	0.935
	EC-RUS (T = 0.859) ^2^	55.8	99.0	96.4	0.640	0.935
	SXGBsite (T = 0.500)	61.6	97.7	95.5	0.600	0.933
	SXGBsite (T = 0.700)	52.1	99.0	96.2	0.618	0.933

^1^ Results excerpted from Yu et al. [7]. ^2^ Results excerpted from Ding et al. [8]. - denotes unavailable.

**Table 9:** Comparison of running time between SXGBsite and EC-RUS (SVM and WSRC) (seconds).

Dataset	SXGBsite ^1^	EC-RUS (SVM) ^2^	EC-RUS (WSRC) ^2^	Dataset	SXGBsite ^1^	EC-RUS (SVM) ^2^	EC-RUS (WSRC) ^2^
ATP	134.5	1746.3	7018.4	$Ca^{2+}$	273.6	6366.5	25627.2
ADP	146.2	4602.8	10940.5	$Mg^{2+}$	290.9	6558.6	31094.1
AMP	118.5	647.5	2298.1	$Mn^{2+}$	124.9	439.5	2806.8
GDP	90.4	284.6	685.8	$Fe^{3+}$	110.6	173.3	1065.9
GTP	92.6	115.8	334.6	$Zn^{2+}$	215.9	4284.6	20220.6
DNA	131.4	4508.5	9083.6	HEME	104.6	3459.9	2940.5

^1^ The PSSM-DCT + PSA feature of SXGBsite is 183-D. ^2^ The PSSM-DCT + PSA feature of EC-RUS (SVM) is 143-D. SVM, support vector machine; WSRC, weighted sparse representation based classifier.

**Table 10:** SXGBsite (average of 10 replicate experiments) compared with the existing methods on the PDNA-41 independent test set.

Predictor	SN (%)	SP (%)	ACC (%)	MCC	AUC
BindN ^1^	45.64	80.90	79.15	0.143	-
ProteDNA ^1^	4.77	99.84	95.11	0.160	-
BindN + (*FPR* ≈ 5%) ^1^	24.11	95.11	91.58	0.178	-
BindN + (*Spec* ≈ 85%) ^1^	50.81	85.41	83.69	0.213	-
MetaDBSite ^1^	34.20	93.35	90.41	0.221	-
DP-Bind ^1^	61.72	82.43	81.40	0.241	-
DNABind ^1^	70.16	80.28	79.78	0.264	-
TargetDNA (*Sen* ≈ *Spec*) ^1^	60.22	85.79	84.52	0.269	-
TargetDNA (*FPR* ≈ 5%) ^1^	45.50	93.27	90.89	0.300	-
EC-RUS (DNA) (*Sen* ≈ *Spec*) ^2^	61.04	77.25	76.44	0.193	-
EC-RUS (DNA) (*FPR* ≈ 5%) ^2^	27.25	97.31	94.58	0.315	-
SXGBsite (*Sen* ≈ *Spec*)	60.35	85.94	84.67	0.272	0.825
SXGBsite (*FPR* ≈ 5%)	35.01	95.01	92.03	0.265	0.825

^1^ Results excerpted from Hu et al. [61]. ^2^ Results excerpted from Shen et al. [44]. - denotes unavailable.
